# Clinical efficacy of “Sancai therapy” for hyperplasia of the mammary glands: A systematic review and meta-analysis

**DOI:** 10.1097/MD.0000000000035989

**Published:** 2023-11-10

**Authors:** Hua Zhang, Zhuye Zhang, Wenhong Li, Jiaxuan Ren, Jianlin Wu

**Affiliations:** a Shandong University of Traditional Chinese Medicine, Jinan, Shandong, China.

**Keywords:** clinical efficacy, hyperplasia of the mammary glands (HMG), meta-analysis, Sancai therapy

## Abstract

**Background:**

Hyperplasia of the mammary glands (HMG) is the most prevalent breast disease and can become malignant if left untreated. Although “Sancai therapy” has been widely used to treat HMG, its efficacy has not yet been systematically reviewed. This study aims to systematically evaluate the clinical efficacy of Sancai therapy for HMG treatment and provide a clinical basis for its future use.

**Methods:**

PubMed, Cochrane Library, Web of Science, EMBASE, CNKI, CBM, VIP, and Wanfang databases were reviewed for related data collection. Chinese and English databases were searched for randomized controlled trials on Sancai therapy for HMG. The retrieval date was February 27, 2023. Exclusion criteria: (1) Non-HMG patients; (2) case reports, literature reviews, animal experiments, systematic reviews; and (3) full text could not be obtained. Data obtained after literature screening were imported into the RevMan 5.4.1 software for meta-analysis, and the included literature was assessed for methodological quality using the “bias risk assessment” tool within the software.

**Results:**

The meta-analysis included 11 studies. Compared to the control group, the Sancai therapy treatment group exhibited an overall increased efficacy (relative risk = 1.36, 95% confidence interval [CI] [1.18, 1.58], *P* < .0001), an increased cure rate (relative risk = 3.74, 95% CI [1.70, 8.25], *P* = .001), a significant improvement in breast pain (standard mean difference = −2.68, 95% CI [−3.41, −1.96], *P* < .00001), and a reduction in breast masses (standard mean difference = −2.87, 95% CI [−3.75, −1.99], *P* < .00001).

**Conclusion:**

Sancai therapy significantly improved the overall efficacy, cure rate, and breast pain and reduced breast mass compared with the control groups. However, further large-sample, high-quality, double-blind randomized controlled trials are required to increase the level of evidence.

**Protocol registration number:**

INPLASY202380124.

## 1. Introduction

Hyperplasia of the mammary glands (HMG) is a noninflammatory, benign breast condition in which structural abnormalities are caused by varying degrees of hyperplasia and subinvolution of the mammary parenchyma and stroma.^[1]^ The principal clinical symptoms of HMG include breast pain and breast masses. Recent studies have shown that HMG is the most prevalent female breast disease, accounting for over 70% of all breast disease cases.^[2]^ Although HMG is a benign disease, its severity increases over time. Studies have shown that it is closely associated with the development of breast cancer, with a malignant transformation rate of 1.25% to 50%.^[3]^ Therefore, effective treatment of HMG is essential for the prevention of breast cancer. Modern medical treatments for HMG primarily consist of hormone or endocrine therapy. Although these treatments provide short-term symptom relief, they have significant side effects and complications. For example, the commonly used endocrine drug tamoxifen increases the risk of abnormal vaginal bleeding, endometrial hyperplasia, uterine fibroids, and endometrial cancer, with a high recurrence rate after drug discontinuation.^[4,5]^

In traditional Chinese medicine (TCM) HMG is classified in the “breast lump” category. TCM traditionally treats breast lumps using methods such as herbal compounding, acupuncture and moxibustion, and massage.^[6,7]^ However, TCM treatment can be lengthy, with low patient compliance. Therefore, TCM is often used as a complementary therapy, and clinicians frequently employ a combination of TCM and Western medicine.

Sancai therapy is a treatment methodology that combines TCM disease identification with Western medical diagnosis. TCM methods are integrated with modern technologies, such as acupoint guidance, smart biowaves, and infrared radiation, to determine a treatment plan. It follows the principle of treating symptoms acutely and the root cause chronically to achieve successful treatment.^[8]^ A growing number of clinical studies have demonstrated satisfactory therapeutic effects of Sancai therapy for the treatment of HMG.^[9]^ However, individual clinical trials have small sample sizes, which limit their ability to provide strong evidence-based support for clinical practice. To address this issue, we conducted a systematic evaluation of randomized controlled trials (RCTs) and performed a meta-analysis based on the objective clinical requirements. This is the first study of its kind to assess the clinical efficacy of Sancai therapy for HMG and to provide reliable evidence to support its use in clinical practice.

## 2. Materials and methods

### 2.1. Study inclusion criteria

#### 2.1.1. Study type.

RCTs published in Chinese and English language were included.

The purpose of this study aimed to evaluate the clinical efficacy of Sancai therapy for the treatment of HMG. As all the data used in this systematic review and meta-analysis have been published, no institutional approval was required.

#### 2.1.2. Study participants.

Patients were diagnosed with HMG according to clinical diagnostic criteria without restrictions based on age, gender, race, or disease duration.

#### 2.1.3. Interventions.

The control group was treated with the patent TCM drugs alone, TCM decoctions, Western medicine, or a combination of TCM and Western medicine. The treatment group received Sancai therapy alone or in combination with other control therapies.

#### 2.1.4. Outcome indicators.

The outcome indicators were the overall efficacy, clinical cure rate, breast pain score, and breast mass score.

### 2.2. Study exclusion criteria

Non-RCTs, theoretical studies, duplicate studies, studies with invalid data or whose full texts were unavailable, and studies that did not mention the baseline comparability of the treatment and control groups were excluded.

### 2.3. Search strategy

We searched the Chinese (CNKI, VIP, Wanfang Data, and SinoMed) and English (PubMed, Web of Science, EMBASE, and Cochrane Library) databases from the time of database establishment until February 27, 2023. For Chinese databases, advanced searches of journal articles were performed using the search terms “乳腺增生” and “三才.” For English databases, we searched for articles using the search terms “Human Mammary Gland,” “Hyperplasias,” and “Sancai.”

### 2.4. Quality evaluation criteria

Two investigators used the “risk of bias assessment” tool in RevMan 5.4.1 software to evaluate the methodological quality of the included articles, and assessed them as “low risk,” “unclear,” or “high risk.” The assessments included random sequence generation, allocation concealment, blinding of participants and personnel, blinding of outcome assessment, incomplete outcome data, and selective reporting of study results.

### 2.5. Diagnostic criteria

Patients were diagnosed with HMG based on the criteria outlined in the “TCM Disease Diagnostic and Therapeutic Criteria” and “Diagnosis and Treatment of HMG” issued by the National Administration of Traditional Chinese Medicine, and the “Diagnostic Criteria for HMG” issued by the Mammary Gland Disease Committee of the Chinese Association of Traditional Chinese Medicine and Surgery, or through clinical examinations such as breast ultrasonography or mammography.

### 2.6. Literature screening and data extraction

Article screening was conducted in 3 stages. First, all retrieved records were imported into the NoteExpress software for screening. Second, the titles and abstracts were read to exclude those that did not meet the inclusion criteria. Finally, the full texts of the remaining articles were read to identify those that met the inclusion criteria. The primary author, year of publication, patient age, sample size, intervention, control treatment, treatment duration, and outcome indicators were extracted from the included articles. This procedure was conducted simultaneously and independently by 2 investigators and cross-checked, compared, and analyzed. Any ambiguous articles or data were discussed and resolved by a third investigator.

### 2.7. Data analysis

The included articles underwent data analysis using RevMan 5.4.1 software, in accordance with the PRISMA guidelines. The relative risk (RR) of binary variables was expressed as the effect size with a 95% confidence interval (CI). Continuous variables were expressed as standardized mean difference (SMD) with 95% CI. For studies with multistage reporting, only results from the last stage were selected. Study heterogeneity was evaluated using the *I*^2^ test. A *P* > .1 and *I*^2^ ≤ 50% indicated good homogeneity among studies, and the fixed-effect model was used. When *P* ≤ .1 and *I*^2^ > 50% indicated significant heterogeneity among studies, and the random-effects model was used. In cases of high heterogeneity, the source was examined through subgroup or sensitivity analyses (individual exclusion of articles). Additionally, when at least 10 articles were included as outcome indicators, funnel plots were constructed to test for publication bias.

## 3. Results

### 3.1. Search results

**Figure 1.**
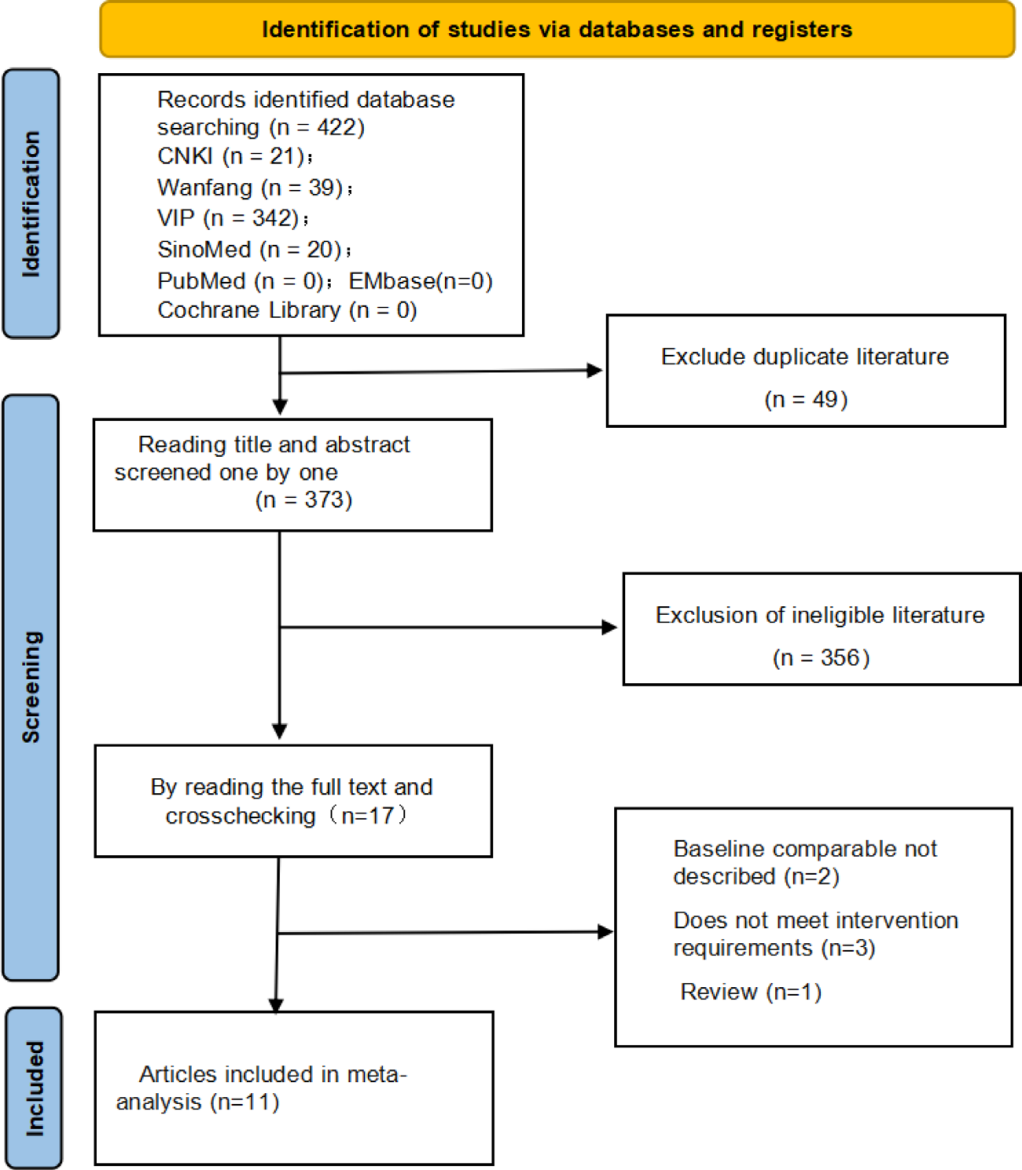
Flowchart outlining the literature search process.

The initial search yielded 422 articles, which were reduced to 373 after eliminating duplicates using NoteExpress. After reading titles and abstracts, 356 articles were excluded. Finally, 11 articles met the inclusion criteria after reading the full text. The article screening process is illustrated in Figure 1.

### 3.2. Characteristics of included studies

Eleven studies^[10–20]^ involving 1077 patients (536 in the control group and 541 in the treatment group) were included in this meta-analysis. All included studies were RCTs conducted in Chinese. The general characteristics of the included articles are listed in Table 1.

**Table 1:** General characteristics.

Authors/year	T cases	C cases	T intervention	C intervention	Outcome indicators	Randomization method
Hu et al^[10]^ 2023	50	50	C + Sancai therapy	Rupisanjie capsules	①②③④	Random groups
Liu et al^[12]^ 2021	42	42	C + Sancai therapy	Comprehensive care	③④	Random number table
Liu et al^[18]^ 2016	60	60	C + Sancai therapy	Vitamin E	①②③④	Random number table
Luo et al^[11]^ 2020	100	100	C + Sancai therapy	Rupisanjie capsules	①②③④	Allocation concealment
Li et al^[20]^ 2021	39	39	Graded Sancai therapy	Xiaoyao tablets	③④	Random numbers
Gao et al^[13]^ 2008	68	63	Graded Sancai therapy	Rupixiao tablets	①②	Only mentions randomization
Luo et al^[19]^ 2022	20	20	C + Sancai therapy	Xiaoyaosan + tamoxifen tablets	①③	Random number table
Guo et al^[14]^ 2022	50	50	C + Sancai therapy device	Rukuaixiao tablets	③④	Only mentions randomization
Bai et al^[15]^ 2016	40	40	C + Sancai therapy device	Xiaoyaosan	③④	Random numbers
Huang et al^[17]^ 2020	32	32	C + Sancai therapy device	Xiaojin pills	①③④	Randomized, double-blind
He et al^[16]^ 2020	40	40	C + Sancai therapy	Breast catheterization	①②③④	Random number table

Note: C = control group, T = treatment group, ① = overall efficacy; ② = cure rate; ③ = breast pain score; ④ = breast mass score.

### 3.3. Methodological quality evaluation

**Figure 2.**
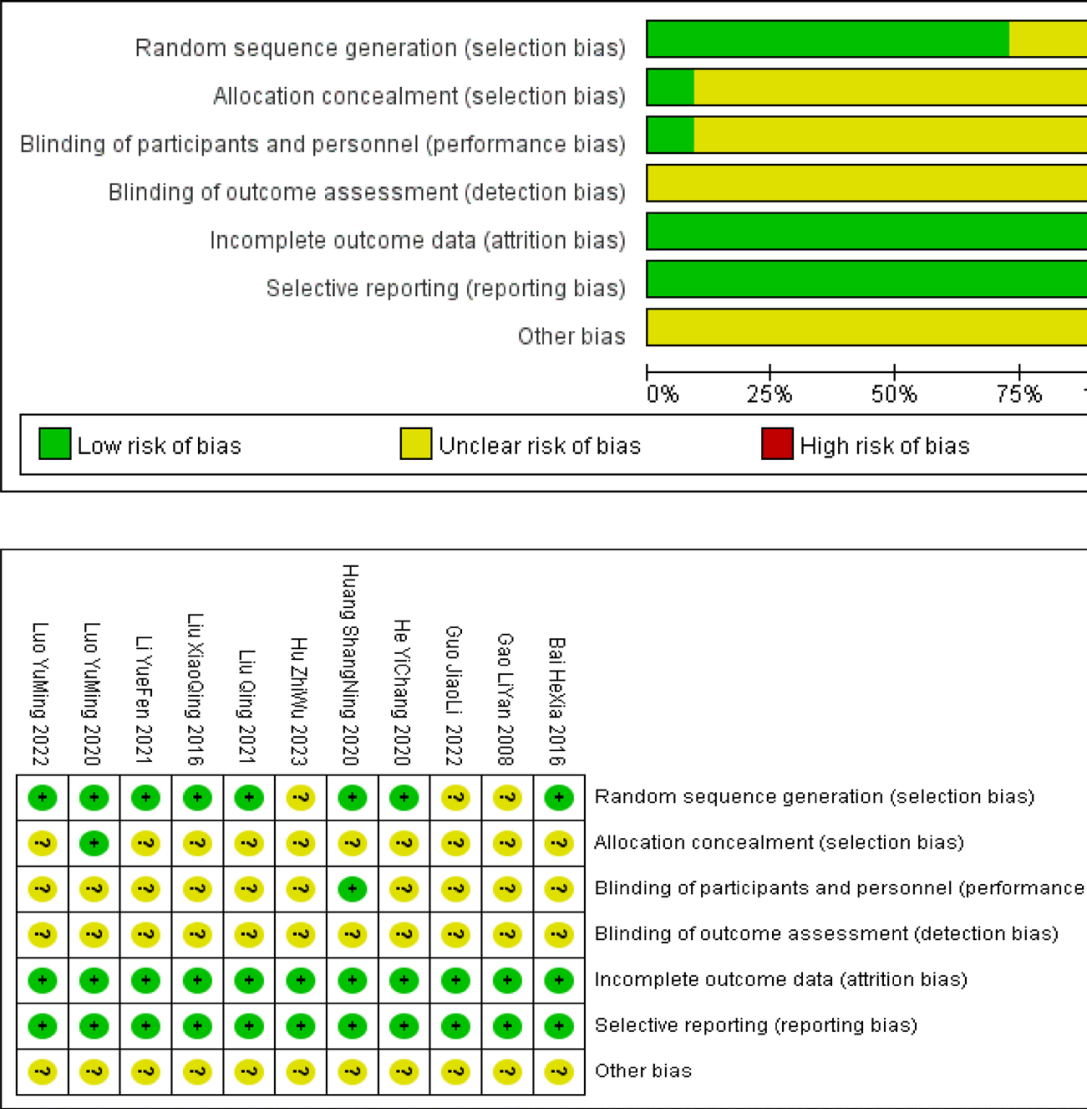
Risk of bias assessment for the included articles.

Eleven RCTs^[10–20]^ were included in this study. For random sequence generation, 6 studies^[12,15,16,18–20]^ used the randomized number table method, 1 study^[11]^ used the randomized envelope method, and 1 study^[17]^ used the randomized double-blind method. All of these methods were considered “low risk.” However, the remaining 3 studies^[10,13,14]^ were all considered to have “unclear” risk as 2 of these studies^[13,14]^ only mentioned randomization and 1 study^[10]^ mentioned random groups but did not specify the grouping method. Allocation concealment was evaluated, and it was found that 1 study^[11]^ used envelope randomization, which was considered “low risk,” while the other studies did not mention it, which was considered “unclear” risk. One study^[17]^ mentioned a double-blind design, which was considered “low risk,” whereas none of the other studies mentioned implementation of blinding, which was considered “unclear” risk. Blinding of the outcome assessment was not mentioned in any of the studies and was considered an “unclear” risk. All studies were rated as “low risk” for incomplete outcome data and selective reporting, while all other biases were rated as “unclear” risk. The results are shown in Figure 2.

### 3.4. Meta-analysis results

#### 3.4.1. Meta-analysis of overall efficacy.

**Figure 3.**
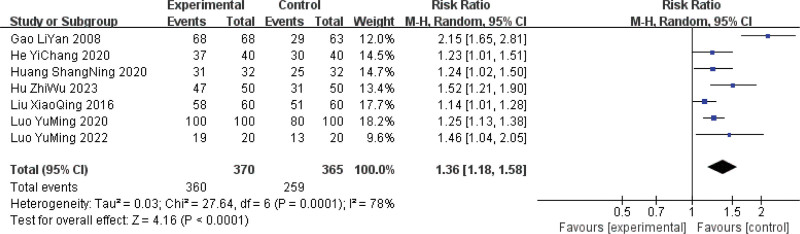
Meta-analysis of the overall efficacy of Sancai therapy for treating HMG. HMG = hyperplasia of the mammary glands.

Seven studies^[10,11,13,16–19]^ reported overall efficacy in 735 patients (370 in treatment groups and 365 in control groups). There was a high heterogeneity among the studies (*P* = .0001, *I*^2^ = 78%). Analysis using a random-effects model showed that the overall efficacy was better in the treatment group than in the control group; the difference was statistically significant (RR = 1.36, 95% CI [1.18, 1.58], *P* < .0001) (Fig. 3).

#### 3.4.2. Meta-analysis of cure rate.

**Figure 4.**
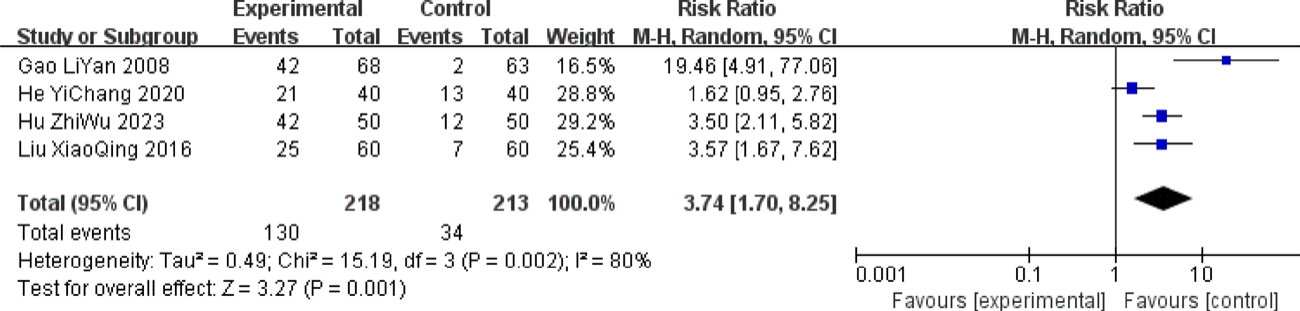
Meta-analysis of the cure rate of Sancai therapy for HMG. HMG = hyperplasia of the mammary glands.

Across 4 studies,^[10,13,16,18]^ cure rates after treatment were reported in 431 patients, with 218 in the treatment groups and 213 in the control groups. There was a high heterogeneity among the studies (*P* = .002, *I*^2^ = 80%). An analysis using a random-effects model showed that the cure rate was significantly higher in the treatment group than in the control group (RR = 3.74, 95% CI [1.70, 8.25], *P* = .001) (Fig. 4).

#### 3.4.3. Meta-analysis of breast pain score.

**Figure 5.**
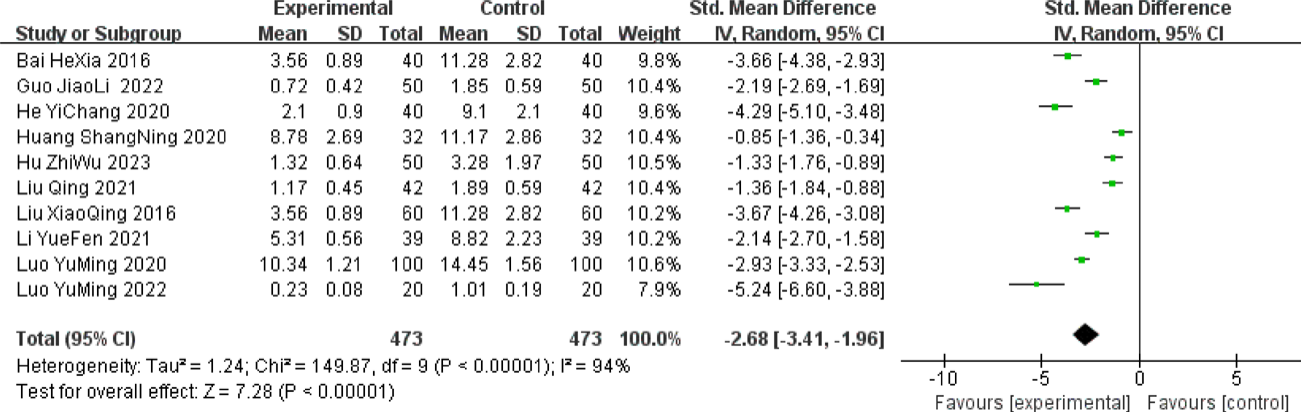
Meta-analysis of breast pain score following Sancai therapy for HMG. HMG = hyperplasia of the mammary glands.

Ten of the included studies^[10–12,14–20]^ reported post-treatment breast pain scores in 946 patients, with 473 in the treatment groups and 473 in the control groups. There was high heterogeneity among the studies (*P* < .00001; *I*^2^ = 94%). Analysis using a random-effects model showed that the pain scores in the treatment group were significantly better than those in the control group (SMD = −2.68, 95% CI [−3.41, −1.96], *P* < .00001) (Fig. 5).

#### 3.4.4. Meta-analysis of breast mass score.

**Figure 6.**
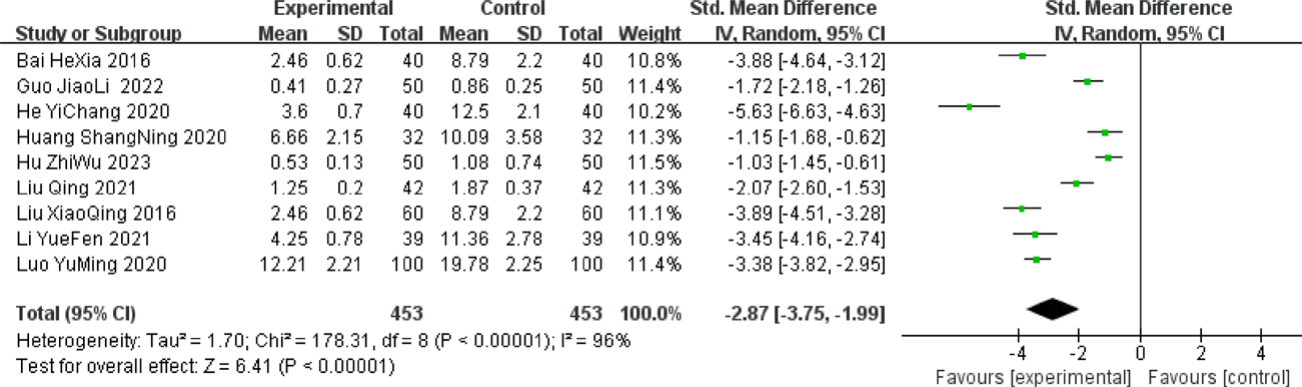
Meta-analysis of the breast mass score following Sancai therapy for HMG. HMG = hyperplasia of the mammary glands.

Nine studies^[10–12,14–18,20]^ reported post-treatment breast mass scores in 906 patients (453 in the treatment groups and 453 in the control groups). There was high heterogeneity among the studies (*P* < .00001; *I*^2^ = 96%). Analysis using a random-effects model showed that the breast mass scores in the treatment group were significantly better than those in the control group (SMD = −2.87, 95% CI [−3.75, −1.99], *P* < .00001) (Fig. 6).

### 3.5. Sensitivity analysis

**Figure 7.**
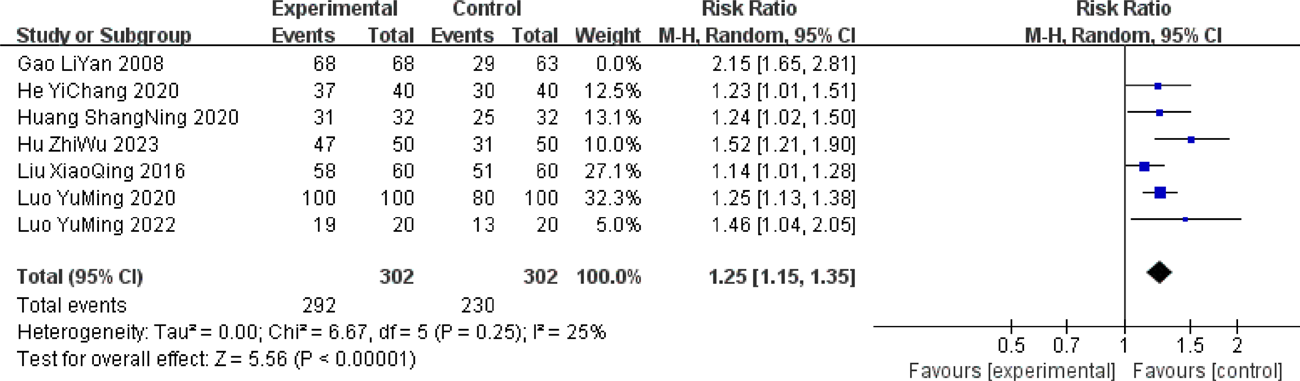
Sensitivity analysis of the overall efficacy of Sancai therapy in treating HMG. HMG = hyperplasia of the mammary glands.

There was high heterogeneity in all 4 outcome indicators, and owing to the small number of included articles, a sensitivity analysis (individual exclusion of articles) was used to identify the sources of heterogeneity. In terms of overall efficacy, heterogeneity was significantly reduced after excluding one study compared to the original results (*P* = .25, *I*^2^ = 25%) but remained statistically significant (RR = 1.27, 95% CI [1.19, 1.36], *P* < .00001), indicating the stability of the meta-analysis results (Fig. 7). For the remaining 3 outcome indicators, the heterogeneity and statistical significance did not change significantly compared with the original results after the exclusion of individual articles, indicating the stability of the meta-analysis results. None of the reviewed articles showed significant sources of heterogeneity, which may be due to clinical heterogeneity.

### 3.6. Publication bias

**Figure 8.**
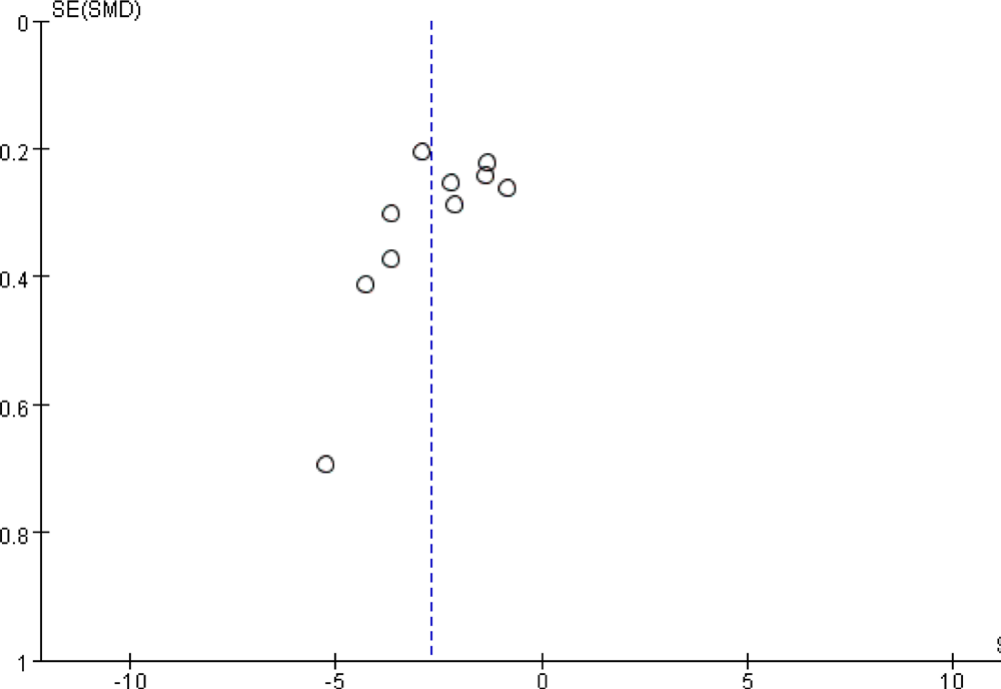
Funnel plot of breast pain scores from included articles.

Breast pain scores were reported in 10 or more articles; therefore, a funnel plot was constructed to test for publication bias. The scattered points were distributed asymmetrically on both sides of the funnel plot (Fig. 8), indicating a degree of publication bias.

## 4. Discussion

The pathogenesis of HMG is complex and, according to modern medicine, is typically attributed to the dysregulation of blood estrogen and progesterone levels, along with their receptors.^[7]^ However, in TCM, the pathogenesis of HMG is primarily attributed to liver stagnation, qi stagnation, spleen disorders, dysregulation of thoroughfare and conception vessels, phlegm coagulation, and blood stasis blocking breast channels. The site of disease is closely associated with the liver, spleen, and kidney.^[21,22]^ Recent studies have shown that the incidence of HMG is increasing each year, coinciding with increased stress levels in various aspects of women’s lives and work, making it the most common disease affecting the breasts of women.^[23,24]^ As individual Western or TCM treatments are no longer sufficient to meet the clinical requirements, it is important to investigate more effective and safer treatment methods to improve the prognosis of patients with HMG.

This is the first meta-analysis to evaluate the clinical efficacy of Sancai therapy for HMG. Eleven studies involving 1077 patients were included in the analysis, thereby expanding the sample size and improving the strength of the clinical evidence. The results indicated that Sancai therapy was more effective than the control group in improving the overall efficacy, cure rate, degree of breast pain, and number of breast lumps. These findings demonstrated that Sancai therapy has significant clinical efficacy in the treatment of HMG.

The Sancai concept was first mentioned in the I Ching: “The Way of Heaven, the Way of Man, and the Way of Earth, and each embraced three powers.”^[25]^ In clinical practice, Sancai is most commonly applied to acupuncture and moxibustion. Sancai therapy is a targeted treatment based on the fundamental theory of acupoint distribution and evidence-based treatment principles. It involves the prescription of specific acupoints in conjunction with electromagnetic data and infrared phototherapy for local physical therapy.^[26]^ Treatment primarily focuses on 3 strategies targeting the causes of HMG. First, a low-frequency pulse probe is applied to designated acupoints, generating an effective electric field that can unblock the mammary meridians; regulate the functions of the liver, spleen, and kidney; relieve liver stress; strengthen the spleen and kidney; and reinforce the head area. This process helps regulate hormone levels and maintain homeostasis in the body.^[8]^ Second, the infrared light is directed at the breast, and its energy penetrates deep into the breast tissue. This accelerates lymphatic flow, improves local blood circulation, promotes tissue metabolism, and enhances the local nutrient supply. Consequently, blood circulation is stimulated, relieving blood stasis, dispersing nodules, and alleviating swelling and pain.^[26,27]^ Third, the magnetic resonance in the probe acts on the corresponding acupoints in the breast, regulating the enzyme activity in the body. This subsequently affects the paramagnetic state of enzymatic reactions, which helps regulate the endocrine system, reduce inflammation, and relieve pain.^[16]^ Sancai therapy is used to cure disease by regulating the body’s “self-balancing” mechanism, which is consistent with the principles of modern medicine. Through this mechanism the nervous system, immune system, endocrine system, and cytokine network interact to maintain homeostasis.^[8]^

Despite the consistent results, this study has some limitations. All the included articles were in Chinese, and some studies had small sample sizes. Additionally, the quality of the articles varied, with some studies failing to comprehensively report on randomization methods, allocation concealment, and implementation of blinding methods. This limits the credibility of our results. The diversity of the diagnostic and efficacy determination criteria among the included articles may have also contributed to the high heterogeneity observed in the study results. Furthermore, well-reported safety and long-term efficacy data are lacking in the included literature. Therefore, the safety and long-term efficacy of Sancai therapy for the treatment of HMG remain unclear.

In conclusion, Sancai therapy can significantly improve the clinical outcomes of patients with HMG by increasing overall efficacy and cure rate, relieving breast pain, and reducing breast lumps. However, to establish the efficacy and safety of Sancai therapy for the treatment of HMG, more high-quality double-blind RCTs with larger sample sizes are needed. Additionally, clinical diagnostic and efficacy criteria should be developed and standardized, and safety and long-term efficacy reports should be published to provide more evidence for the use of Sancai therapy in clinical practice. The abbreviations of this article are shown in Table 2.

**Table 2:** Abbreviations.

Abbreviations	Full name
HMG	hyperplasia of the mammary glands
RCTs	randomized controlled trials
RR	relative risk
CI	confidence interval
SMD	standard mean difference
TCM	traditional Chinese medicine

## Acknowledgments

We thank all the authors of the study and Professor Wu Jianlin for their valuable comments on this article. Thank you for the literature help provided by the library of Shandong University of traditional Chinese Medicine. We also thank CNKI and Web of Science for their language assistance during the preparation of this manuscript.

## Author contributions

**Conceptualization:** Hua Zhang, Zhuye Zhang.

**Data curation:** Hua Zhang, Zhuye Zhang.

**Formal analysis:** Hua Zhang, Zhuye Zhang, Wenhong Li, Jiaxuan Ren.

**Funding acquisition:** Jianlin Wu.

**Methodology:** Hua Zhang, Wenhong Li, Jiaxuan Ren.

**Software:** Zhuye Zhang, Wenhong Li, Jiaxuan Ren.

**Supervision:** Jianlin Wu.
